# Oxytocin Enhances Social Persuasion during Hypnosis

**DOI:** 10.1371/journal.pone.0060711

**Published:** 2013-04-05

**Authors:** Richard A. Bryant, Lynette Hung

**Affiliations:** School of Psychology, University of New South Wales, Sydney, Australia; Ecole Normale Supérieure, France

## Abstract

It has long been argued that hypnosis cannot promote behaviors that people will not otherwise engage in. Oxytocin can enhance trust in others, and may promote the extent to which a hypnotized person complies with the suggestion of a hypnotist. This double-blind placebo study administered oxytocin or placebo to high hypnotizable participants (N = 28), who were then administered hypnotic suggestions for socially unorthodox behaviors, including swearing during the experiment, singing out loud, and dancing in response to a posthypnotic cue. Participants who received oxytocin were significantly more likely to swear and dance than those who received the placebo. This finding may be interpreted in terms of oxytocin increasing social compliance in response as a function of (a) increased trust in the hypnotist, (b) reduced social anxiety, or (c) enhanced sensitivity to cues to respond to experimental expectations. These results point to the potential role of oxytocin in social persuasion.

## Introduction

Hypnotized people are able to respond in ways that are highly incongruent with normal patterns of perception, cognition, and behavior. A hypnotized person may experience for suggested events, rigidity of body parts, or anesthesia in response to painful stimuli. Accordingly, hypnosis has intrigued researchers since populated by Freud [1] and Janet [2] over 100 years ago. Hypnotizability (i.e., one's capacity to respond to hypnotic suggestion) is normally distributed in the population, with approximately 15% being low hypnotizable, 70% being medium hypnotizable, and 15% being high hypnotizable [3]. Further, one's capacity for hypnotic responding is very stable across time, with evidence that it remains consistent over 25 years (*r* = 0.71) [4].

It has traditionally been argued that hypnotized people will not respond to hypnotic suggestions that are contrary to behaviors they would ordinarily engage in [5]. Prompted by cases of people allegedly performing criminal acts during hypnotic suggestion [6], earlier research studied the capacity of hypnosis to prompt an individual to engage in antisocial or undesirable behavior. Although some earlier work suggested that hypnotized people would engage in undesirable or dangerous acts [7], subsequent studies suggested that these effects could be attributed to social compliance factors that were independent of hypnosis [8].

The current study renews this line of investigation with a novel approach. There is much evidence that hypnotic responding is influenced by the nature of the relationship with the hypnotist, insofar as hypnotic responding is determined by hypnotized participants being motivated by contextual factors to comply with hypnotic suggestions [9]. Consistent with this proposal, hypnotic response is enhanced by increasing participant's motivation [10], and that hypnotic response is moderated by manipulating rapport with the hypnotist [11].

One potential means to enhance responding during hypnosis is to enhance rapport via direct manipulation of oxytocin. Oxytocin has been shown to influence bonding and social affiliation by acting as a neurotransmitter/neuromodulator. Specifically, administering oxytocin enhances a range of social behaviors in animals [12], including maternal nurturing behaviors [13], pair-bonding [14], while antagonists of oxytocin impair bonding [15]. Similarly in humans, oxytocin administration has been shown to enhance trust and prosocial behaviour [16], less amygdala recruitment to social cues [17], detection of affective states in others [18], attention to people's eyes [19], and encoding of positive social memories. Other evidence has accumulated that points to oxytocin playing a more nuanced role than simply enhancing prosocial tendencies. There is evidence that oxytocin facilitates envy [20], ethno-centric prejudice [21], limits trust when information about social partners is unavailable [22], enhances responsivity to aversive social stimuli [23], and boosts mistrust in those with histories of poor attachments [24]. Further, it has been shown that the influence of oxytocin is dependent on contextual cues, such that oxytocin administration prompts men in monogamous relationships to maintain distance from attractive women but this effect was not observed in single men [25]. Following this evidence, it has been suggested that oxytocin may facilitate attention to social cues and the subsequent behavioral response may be modulated by contextual and attitudinal factors [24].

There is evidence that hypnotic response can be increased in low hypnotizable participants by administration of oxytocin relative to placebo [26]. This finding could be attributed to several potential mechanisms. Oxytocin could enhance hypnotic response by reducing anxiety about engaging in hypnotic behavior. Oxytocin has been shown to reduce hypothalamic-pituitary-adrenal axis (HPA) activity [27]. Alternately, on the basis that oxytocin enhances attention to social cues, and under positive conditions prosocial behavior, oxytocin administration may enhance the trust that an individual has in the hypnotist, thereby leading to greater hypnotist response. Accordingly, we hypothesized that oxytocin administration may enhance engagement in socially unorthodox behaviors.

## Materials and Methods

### Participants

Hypnotizability was initially assessed on two occasions prior to the current experiment to ensure that we recruited highly hypnotizable participants. We initially screened 850 university students for hypnotizability levels via a group-administered hynotizability test: the Harvard Group Hypnotizability Scale: Form A [28]. This test comprises a hypnotic induction followed by 12 suggestions (4 motor, 4 challenge, and 4 cognitive suggestions). We employed a 10-item adaption of the self-scored HGSHS:Form A comprising eyes closing, extended arm falling, difficulty lifting arm, difficulty separating interlocked fingers, extended arm difficult to bend, difficulty shaking head “no”, difficulty opening closed eyes, swatting at a hallucinated fly, touching ankle in response to posthypnotic cue, and amnesia for events in hypnosis (a shortened version was employed to comply with time constraints). Participants were then re-assessed on a 10-item version of the individually-administered experimenter-scored Stanford Hypnotic Susceptibility Scale, Form C (SHSS-C) [29]. The SHSS-C is a more rigorous measure of hypnotic susceptibility because (a) it is individually administered, and (b) has a greater proportion of difficult cognitive hypnotic items that require alterations in cognitive experience rather than motor responses. We employed a 10-item adaption of this scale that comprised motor (moving hands apart), challenge (difficulty bending extended arm, difficulty lifting arm), and cognitive (swatting a hallucinated mosquito, hallucinating a taste, dream, age regression to school, anosmia to ammonia, hallucinating a voice, and posthypnotic amnesia). Only participants who scored in the range 7–10 on the SHSS:C were recruited for the study. We selected 28 high hypnotizable participants who scored 7–10 (M = 8.42, SD = 1.14) on the HGSHS:Form A, and 7–10 (M = 8.51, SD = 1.15) on the SHSS:C. We restricted the sample to males because of potential adverse effects of oxytocin administration on pregnancy. We also excluded individuals with reported allergies to preservatives contained in the nasal spray (viz., E216, E218, and chlorobutanol hemihydrate).

### Procedure

This project was approved by the University of New South Wales Human Research Ethics Committee. Following written informed consent, participants were administered a brief medical examination to ensure that there were no contraindications for oxytocin administration (no participants were excluded). Participants abstained from alcohol and caffeine on the day of oxytocin administration, and food and drink (except water) 2 hours before the oxytocin administration. Participants initially rated levels of anxiety and trust about the hypnosis session on 7-point Likert scales (1 = *none*, 7 = *extremely*). Participants then self-administered an intranasal spray of 24 IU oxytocin (n = 15) or placebo (n = 13) that involved four puffs of 3IU in each nostril. Placebo spray involved the same ingredients with the exception of oxytocin (i.e., sorbitol, glycerol, benzyl alcohol, and distilled water). A double-blind methodology was adopted and codes were not released by the chemist until the final experimental session was complete. Forty-five minutes after intranasal administration, participants again rated their anxiety and trust.

Participants were then administered a hypnotic induction comparable to the hypnotic induction used in the SHSS:C. Participants were then administered a number of filler hypnotic items. Participants were then given the suggestion to describe their activities last weekend and during this they would “*feel the urge to swear*”. Participants were then given more two more filler items, and then listened to background music with the instruction to “*feel an urge to sing out loud*”. Prior to cancelling hypnosis, participants were given a posthypnotic suggestion to “*feel the urge to get up from your chair and dance*” when they heard the hypnotist say the phrase, “*Let's take a break*”; this suggestion was combined with a suggestion for posthypnotic amnesia in which they were told that they would not remember why they felt the urge to dance. Hypnosis was then terminated, and an interview was conducted during which the posthypnotic cue was given, then cancelled, and the session terminated.

Two independent raters, in addition to the experimenter, were all blind to the oxytocin/placebo conditions subsequently rated from video recordings the extent to which each item was passed (1 = *none*, 7 = *extremely*). There was very strong reliability between ratings for the singing (*r* = 0.71–.88, *p*<.001), swearing (*r* = 0.98–.99, *p*<.001), and dancing (*r* = 0.86–.93, *p*<.001) responses. Ratings were summed for purposes of analyses.

## Results and Discussion

Participants in the two conditions did not differ in terms of demographic characteristics, or hypnotizability scores. Separate 2 (Condition)×2 (Assessment Time) analyses of variance (ANOVAs) of trust and anxiety ratings prior to and following the spray indicated no significant main or interaction effects (see Table 1).

**Table 1:** Participant Characteristics and Experimental Ratings.

	Oxytocin	Placebo
Age	20.13 (1.45)	19.54 (1.40)
HGHS:A	8.50 (1.14)	8.31 (1.16)
SHSS:C	8.43 (1.08)	8.62 (1.27)
Trust Rating 1	6.32 (0.58)	5.88 (0.89)
Trust Rating 2	6.26 (0.03)	6.00 (0.84)
Anxiety Rating 1	2.60 (1.51)	1.85 (1.14)
Anxiety Rating 2	3.13 (1.96)	1.81 (1.41)
Swearing Rating	6.27 (5.87)	1.95 (3.75)
Singing Rating	3.24 (1.54)	2.59 (1.53)
Dancing Rating	3.10 (2.00)	1.54 (1.69)

*Note.* HGHS:A = Harvard Group Hypnotizability Scale: Form A. SHSS:C = Stanford Hypnotic Suspectibility Scale: Form C. Standard deviations appear in parentheses.

**Figure 1:**
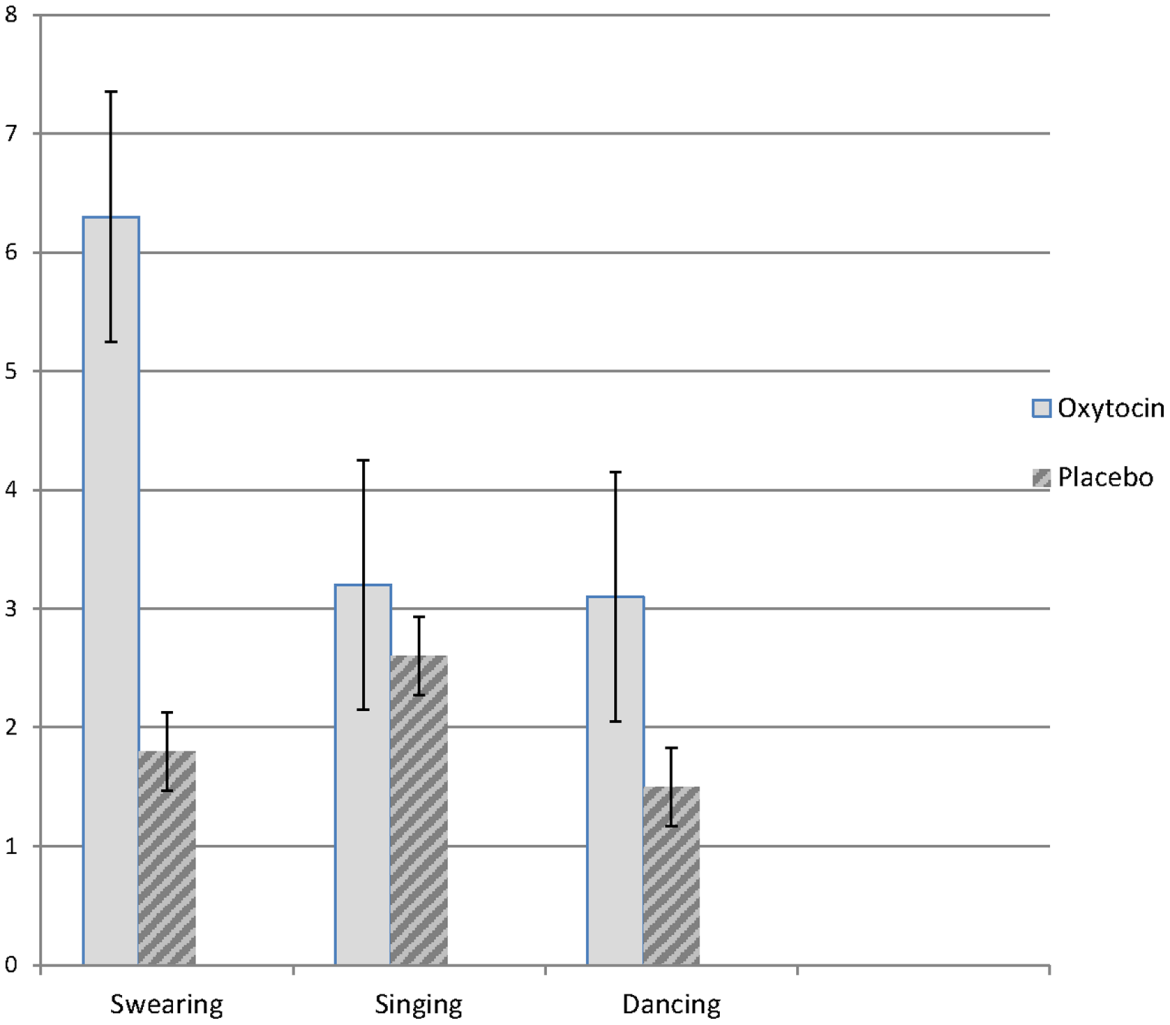
Mean ratings by independent rater of behavioral responses during the hypnosis session. Error bars represent standard errors of the mean.

An independent rated each participant's response to the suggestion for swearing, singing, and spontaneous dancing as pass or fail. Significantly more participants in the oxytocin condition passed the swearing [80% vs 39%; χ^2^(N = 28) = 5.04, p<.05] and dancing [67% vs 23%; χ^2^(N = 28) = 5.32, p<.05] suggestions than those in the placebo condition (but no difference for singing [67% vs 69%; χ^2^(N = 28) = 0.02, p = .89]. Figure 1 demonstrates that participants in the oxytocin condition were rated by the independent raters as swearing [*t* (26) = 2.28, *p*<.05] and dancing [*t* (26) = 2.17, *p*<.05] more than those in the placebo condition.

The noteworthy aspect of this double-blind study is that participants who received oxytocin engaged in swearing and dancing more than those who received the placebo. It is possible that this behavior was facilitated because oxytocin led to reduced anxiety about the social situation which in turn led to a lower threshold for engaging in potentially socially embarrassing behavior; this interpretation accords with evidence that oxytocin decreases anxiety in socially stressful situations [30]. Alternately, participants receiving oxytocin trusted the hypnotist more than those receiving placebo, thereby having greater motivation to participate in these behaviors. Third, recent evidence suggests that oxytocin increases sensitivity to social cues generally [24], [31]. There is much evidence that perceived social expectations of the experimental setting are highly influential in hypnotic response [32], and oxytocin may facilitate sensitivity to these cues.

The finding that oxytocin did not result in altered self-reports of anxiety or trust relative to placebo counters, to some extent, the interpretations that the effects of oxytocin may be attributed to increases in trust or decreases in anxiety. Oxytocin does have anxiolytic effects [33], [34] via dampening of hypothalamic-pituitary-adrenal axis activity [35], and has been shown to reduce social anxiety in experimental settings [30]. Nonetheless, this pattern was not observed in the current study. This may reflect the absence of an effect of oxytocin on anxiety or trust, the possibility that the effect of oxytocin was not perceived by participants, or that participants perceived a demand characteristic to not report differences in ratings over time.

Earlier studies have reported marginal increases in suggestibility following administration of LSD, mescaline, and psilocybin [36]. The effect of most of these drugs has been interpreted in terms of these drugs reducing reality testing, and therefore allowing greater responsiveness to hypnotic suggestions. In contrast, oxytocin does not alter consciousness and hence the observed effect of oxytocin on increasing response to the unorthodox behaviors needs to be explained by a mechanism distinct from reduced reality testing.

We recognize that suggesting to participants that they dance, sing, and swear in an experimental setting may not represent antisocial or undesirable behaviors. There is much evidence to suggest that hypnotized participants reframe the suggested experiences in ways that allow them to construe them as acceptable within the constraints of the experimental framework [11], [37]. Oxytocin may have facilitated sufficient rapport in the experimental setting to permit acceptance of these behaviors as acceptable in light of the experimental demands. More rigorous study would require the participant to engage in explicitly antisocial behaviour, as much as one can ethically permit in research, and also to test this outside the perceived realm of the experimental context.

We note several limitations. To avoid potential complications with undetected pregnancies we excluded female participants, and so we do not know if these findings are applicable to females. We also tested all participants within a hypnotic context, which leaves open the question concerning oxytocin increasing the capacity to influence people to engage in unorthodox behavior outside hypnosis. It is important to note that many studies have replicated hypnotic responsivity studies by heightening the motivation of participants and suggesting that they comply with suggested experiences [38], [39]. The current design does not allow us to delineate between the effects of oxytocin on persuasion in hypnotic and nonhypnotic contexts. An intriguing avenue for future research is to determine the extent to which people may engage in behaviors that they would otherwise not participate in if persuaded under the influence of oxytocin.

These findings suggest that, despite earlier reports that hypnosis will not lead to behaviors normally unacceptable to the participant, oxytocin can facilitate this behavior. One previous study has found that oxytocin does not increase gullibility insofar as participants do not increase trust if the other person is not trusted [40]. Given the evidence that most hypnotic behaviors can be achieved outside hypnosis if the appropriate context is established [10], there is a need to determine the extent, and associated mechanisms, to which oxytocin may enhance social persuasion generally.
